# Supplementary material for: Genome length determination in adeno-associated virus vectors with mass photometry
Source: Mol Ther Methods Clin Dev. 2023 Nov 19;31:101162. doi: 10.1016/j.omtm.2023.101162 (PMC10716024; doi:10.1016/j.omtm.2023.101162)
Supplement: Document S1. Figures S1 and Tables S1–S3 [file mmc1.pdf]

OMTM, Volume *31*

Supplemental information

Genome length determination

in adeno-associated virus vectors

with mass photometry

Cornelia Hiemenz, Nadine Baumeister, Constanze Helbig, Andrea Hawe, Sabrina Babutzka, Stylianos Michalakis, Wolfgang Friess, and Tim Menzen

Table S1. List of AAV samples (cp, capsid particle; vg, viral genome; WT, wild-type). The expected genome length corresponds to the used transgene size. Transgene size was confirmed by sanger sequencing (Eurofins Genomics). Transgene titer was determined by qRT-PCR with ITR-specific primers on a StepOnePlus Real-Time PCR system (Applied Biosystems, Thermo Fisher Scientific). Standard curves were established using ITR-specific primers as described before [1], [2]. Capsid titer as well as content of residual iodixanol were determined by using the Stunner instrument (Unchained Labs); (*) Note that the high residual iodixanol content in the AAV No. 5 sample most likely led to an underestimation of capsid titer.

**No.**	**Serotype**	**Transgene size [b]**	**Transgene titer [vg/mL]**	**Capsid titer [cp/mL]**	**Residual iodixanol [% (v/v)]**
**1**	AAV9 WT	0 (empty capsid)	-	2.91×10^{14}	12.0
**2**	AAV9 WT	3793	2.55×10^{14}	8.70×10^{14}	3.5
**3**	AAV9 WT	4142	3.14×10^{14}	9.62×10^{14}	4.2
**4**	AAV9 WT	4504	3.30×10^{14}	1.69×10^{15}	5.4
**5**	AAV9 WT	4596	6.88×10^{13}	5.43×10^{12}*	28.0
**6**	AAV9 WT	4658	6.73×10^{13}	3.50×10^{14}	13.0
**7**	AAV2 WT	4658	2.56×10^{13}	1.81×10^{14}	10.0

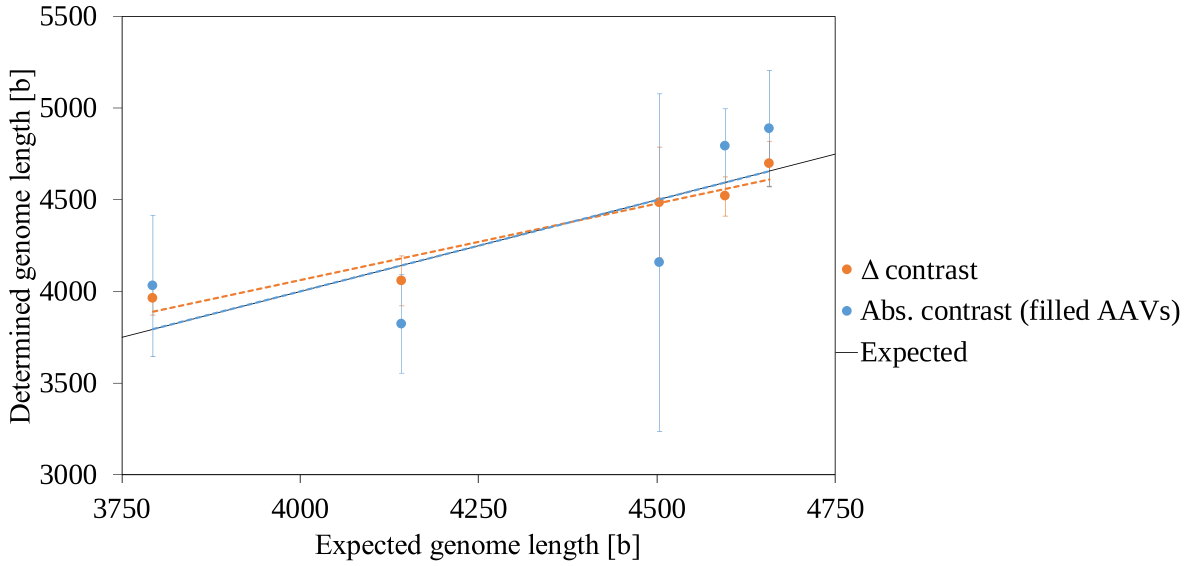

Figure S1. Genome length determination according to approach 2, when High Molecular Weight standard was used as reference point: Genome lengths (in bases) determined for five AAV samples using either a calibration curve based on the contrast difference between High Molecular Weight standard and filled AAVs of known genome length (Δ contrast) or a calibration curve based on the absolute contrast values of AAVs of known genome length (Abs. contrast (filled AAVs)); Error bars represent standard deviation (n=3).

Table S2. Genome lengths of the AAV samples No. 2-6 determined using intact AAVs as calibration material (approach 2), presented as mean of the three measurement days with SD, %CV and accuracy. On each measurement day, n=3 to 4 measurements were performed. In this case, the ratiometric contrast difference (Δ contrast) was calculated between the filled AAV and the High Molecular Weight standard (instead of empty AAV).

		**AAV No. 2**	**AAV No. 3**	**AAV No. 4**	**AAV No. 5**	**AAV No. 6**
**Expected genome length [b]**		3793	4142	4504	4596	4658
**Δ contrast calibration**	Genome length ± SD [b]	3963 ± 91	4057 ± 137	4486 ± 300	4518 ± 107	4697 ± 123
	Accuracy	104%	98%	100%	98%	101%
	%CV	2.3%	3.4%	6.7%	2.4%	2.6%
**Abs. contrast (filled AAVs) calibration**	Genome length ± SD [b]	4030 ± 387	3823 ± 271	4156 ± 921	4794 ± 203	4887 ± 317
	Accuracy	106%	92%	92%	104%	105%
	%CV	9.6%	7.1%	22.2%	4.2%	6.5%

Table S3. Molecular masses or genome lengths of the molecules used for MP calibration; MW marker, molecular weight marker. *When ssDNA plasmids were applied for calibration, the calibration curve was fitted through the zero point.

Protein-based calibration curve

Molecule	Urease (monomer)	Urease (dimer)	Thyroglobulin	AAV No. 1 (empty capsid)
Molecular mass [kDa]	272	545	670	3700

RNA-based calibration curve

NW marker	RiboRuler High Range RNA ladder							
Fragment length [b]	200	500	1000	1500	2000	3000	4000	6000

ssDNA-based calibration curve

Plasmid	Zero point*	ΦX174 Virion	M13 mp18
Genome length [b]	0	5386	7249

dsDNA-based calibration curve

MW marker	Low DNA mass ladder					
Fragment length [b]	100	200	400	800	1200	2000

AAV-based calibration curves

AAV	AAV No. 2	AAV No. 3	AAV No. 4	AAV No. 5	AAV No. 6
Genome length [b]	3793	4142	4504	4596	4658

REFERENCES

[1] C. Aurnhammer *et al.*, “Universal real-time PCR for the detection and quantification of adeno-associated virus serotype 2-derived inverted terminal repeat sequences,” *Hum. Gene Ther. Methods*, 2012, doi: 10.1089/hgtb.2011.034.

[2] S. D’Costa *et al.*, “Practical utilization of recombinant AAV vector reference standards: focus on vector genomes titration by free ITR qPCR,” *Mol. Ther. - Methods Clin. Dev.*, vol. 3, no. January, p. 16019, 2016, doi: 10.1038/mtm.2016.19.
