# Supplementary material for: Genome length determination in adeno-associated virus vectors with mass photometry
Source: Mol Ther Methods Clin Dev. 2023 Nov 19;31:101162. doi: 10.1016/j.omtm.2023.101162 (PMC10716024; doi:10.1016/j.omtm.2023.101162)
Supplement: Document S2. Article plus supplemental information [file mmc2.pdf]

Molecular Therapy
Methods & Clinical Development
Original Article

Genome length determination in adeno-associated virus vectors with mass photometry

Cornelia Hiemenz,[1] Nadine Baumeister,[1,3] Constanze Helbig,[1] Andrea Hawe,[1] Sabrina Babutzka,[2] Stylianos Michalakis,[2] Wolfgang Friess,[3] and Tim Menzen[1]

[1]Coriolis Pharma Research GmbH, 82152 Martinsried, Germany; [2]Department of Ophthalmology, University Hospital, LMU Munich, 80336 Munich, Germany; [3]Department of Pharmacy, Pharmaceutical Technology and Biopharmaceutics, LMU Munich, 81377 Munich, Germany

Recombinant adeno-associated viruses (rAAVs) are attractive therapeutic viral vectors for gene delivery. To ensure the efficacy and safety of rAAV-based therapies, comprehensive characterization of the adeno-associated virus (AAV) capsids is essential. Mass photometry (MP) provides the advantage of short analysis times, low sample consumption, and high accuracy of molecular mass determination. Despite having just recently emerged, MP has already been used to characterize AAV genome content and quantify filled/empty capsid ratios. In this study, we explored three approaches for the application of MP to assess genome length in AAVs. In approach 1, genome length in intact AAVs was approximated with good precision (coefficient of variation [%CV] < 2.6%) and accuracy (±5%) by using a straightforward protein-based calibration. In approach 2, genome length was determined even more accurately (±1%, %CV < 2.9%) considering calibration with a set of additional AAVs of different genome length. In approach 3, genome length was assessed after genome release from the capsid by heating in 1% sodium dodecyl sulfate followed by surfactant removal with precision of %CV < 0.7% and accuracy of ±5%. In conclusion, the three developed MP-based approaches are fast, precise, and accurate methods for genome length determination in AAVs, differing in their calibration materials and efforts.

INTRODUCTION

The preclinical and clinical success of recombinant adeno-associated viruses (rAAVs) as therapeutic viral vectors for the treatment of severe human diseases established them as an attractive platform for gene delivery. As of spring 2023, several rAAV products are under review for the treatment of rare genetic disorders, with four rAAV-based therapeutics already approved by the European Medicines Agency (EMA) (https://euclinicaltrials.eu): Luxturna against inherited retinal dystrophy (Spark Therapeutics), Zolgensma against spinal muscular atrophy type 1 (Novartis Europharm), Upstaza against aromatic L-amino acid decarboxylase (AADC) deficiency (PTC Therapeutics), and Roctavian against hemophilia A (BioMarin).

Adeno-associated viruses (AAVs) are small, nonenveloped icosahedral viruses belonging to the parvovirus family with a diameter of approximately 25 nm. The capsids are 60-mers assembled from three viral proteins (VPs), VP1–VP3, at a ratio of ~5:5:50.[1,2] Each capsid contains a single-stranded DNA (ssDNA) genome with a length of up to ~4.7 kb.[3,4] The wild-type (WT) genome comprises (1) the *rep* gene, encoding proteins necessary for viral genome replication and transcription, and (2) the *cap* gene, encoding VP1–VP3, all flanked by the inverted terminal repeats (ITRs) required for replication and packaging.[5,6] In rAAVs used for gene therapy, the *rep* and *cap* genes are replaced by the respective therapeutic gene expression cassette.

There are multiple strategies to generate AAV vectors. The frequently used mammalian cell-based production systems involve triple transfection with three plasmids that encode (1) the expression cassette for the transgene, (2) the AAV *rep* and *cap* genes, and (3) the viral helper functions from adenovirus or herpes simplex virus essential for AAV replication.[7–9] During the virion assembly, rAAVs are generated containing the therapeutic, full-length genome (filled AAVs) but also undesired capsids, which contain no genome (empty AAVs), fragments of the therapeutic genome, host-cell genome, or plasmid impurities.[10,11]

The identity and integrity of the rAAV vector genome are important quality attributes that affect the infectivity, efficacy, and safety of gene therapy products.[12,13] Thus, determining the quality of rAAV vectors by verifying the encapsidated genome length (i.e., the genome's number of bases) is crucial. Overall, the complexity of the viral vectors, consisting of the protein capsid assembly and the nucleic acid genome, often impedes simple, cost-effective, and robust characterization.

Received 16 May 2023; accepted 16 November 2023;
https://doi.org/10.1016/j.omtm.2023.101162.

Correspondence: Tim Menzen, Coriolis Pharma Research GmbH, Fraunhoferstraße 18B, 82152 Martinsried, Germany.
E-mail: tim.menzen@coriolis-pharma.com

This is an open access article under the CC BY license (http://creativecommons.org/licenses/by/4.0/).

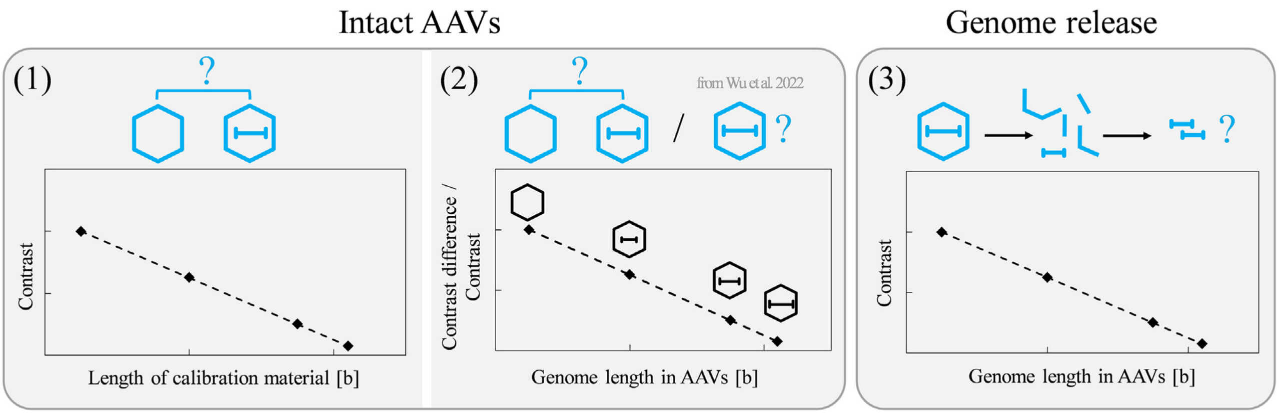

Figure 1. Overview of the three tested approaches for the determination of AAV genome length in number of bases

(1) The contrast difference between empty and filled AAV populations is translated into genome length with the help of a calibration curve on the basis of isolated RNA, dsDNA, ssDNA plasmids, or proteins in approach 1. (2) Approach 2 uses AAVs of known genome length for calibration. Two analysis procedures were compared. Either the contrast difference between empty and filled AAVs is converted into genome length or the contrast value of each AAV is directly converted into genome length according to the published approach of Wu et al.[19] (3) In approach 3, the genome is released from the capsid and the contrast value measured for the released genome is translated to genome length on the basis of a calibration with isolated RNA or ssDNA plasmids.

To assess whether the filled AAV vectors contain a genome of the expected length, several methods are available. Typically, the genome length is verified by gel electrophoresis and Southern blot, after disruption of the AAVs during sample preparation. Alternatively, the genome length is estimated with the help of sedimentation velocity analytical ultracentrifugation (SV-AUC). These methods come with substantial time and material requirements.[14–16] Other instrumentation such as capillary gel electrophoresis systems or automated electrophoresis tools provide a simpler workflow and require less sample material compared with the gel-based methods.[17,18] Recently, charge detection mass spectrometry (CDMS) has been successfully applied to characterize various genome lengths in rAAV8.[18] Although rAAV samples could be analyzed at high throughput and with minimal sample consumption, the access to commercially designed CDMS instruments is currently limited.

Mass photometry (MP) as a novel analytical method opens possibilities for the analysis of the genome content in AAVs. It has been shown that MP is a rapid and effective method to distinguish empty and filled capsids and to quantify the relative abundance of each population.[19–21] MP was even capable to identify partially filled capsids, although with slightly poorer resolution than SV-AUC.[19]

MP measures the molecular mass of individual macromolecules, specifically biomolecules of 40 kDa to 5 MDa in a sample.[22] The local change in reflectivity at a glass-water interface, caused by the attachment of a biomolecule from solution to a coverslip surface, results in a local contrast change. The contrast change for each particle surrounded by a medium of refractive index n_m directly correlates with the particle's volume and refractive index.[23] Consequently, the contrast value is proportional to the molecular mass, assuming that the optical properties of the scatterer do not vary significantly (~1%).[23] The contrast value can be transformed into molecular mass using a calibration with biomolecules of known molecular mass. Hence, MP offers a promising approach to determine the molecular mass of proteins and protein assemblies but also the chain length of nucleic acids, if the corresponding calibration material is used.[24,25] MP convinces by its simplicity and requires only sub-picomolar quantities of the analyzed material.[19,24]

In this study, we addressed the question of how MP can be used to determine the genome length in AAV vectors. Three main approaches for assessing the genome length were compared (Figure 1). In approach 1, we assume that the contrast change between filled and empty AAVs is attributed to the encapsidated genome, so that the contrast difference between these two populations can be translated into genome length using a suitable calibration material. Because the light is scattered at the capsid wall as well as at the encapsidated genome, we assessed the accuracy of genome sizing results applying four types of calibration material: proteins, double-stranded DNA (dsDNA), RNA, and single-stranded circular DNA plasmids (ssDNA plasmids). In approach 2, AAVs of known genome length were used for calibration in two ways: (1) the relation between known genome length and corresponding contrast difference between empty and filled calibration AAVs is applied to obtain the genome length of an unknown AAV sample on the basis of the contrast difference between empty and filled AAV populations, and (2) the absolute contrast values of the analyzed filled AAVs are directly translated into genome length using filled AAVs of known genome length, as previously described by Wu et al.[19] In approach 3, the genome length is not determined from intact AAVs, as in approaches 1 and 2, but after releasing the genome from the capsid and using RNA or ssDNA plasmids for calibration.

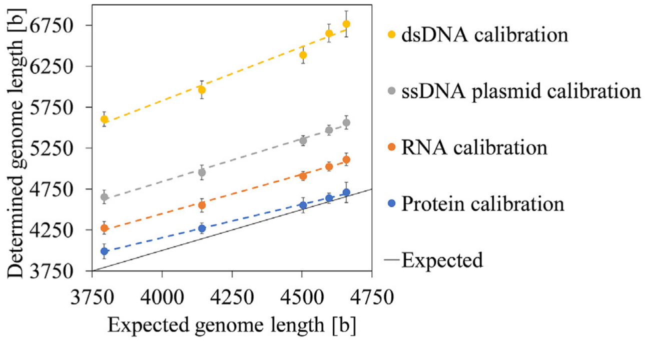

Figure 2. Approach 1

Plot of determined versus expected genome lengths (in number of bases) for five AAV samples. Protein- and nucleic acid-based calibration materials were used. Error bars represent SD (n = 3).

RESULTS

Genome length determination in intact AAVs

Approach 1: Conversion of contrast difference between filled AAVs and empty AAVs in genome length on the basis of various calibration materials

For approach 1, the contrast difference between the peaks of filled and empty AAVs 2–6 (see Table S1) was converted to genome length in bases by applying a calibration curve on the basis of dsDNA, ssDNA plasmids, RNA, or proteins. We found a strong positive correlation for all calibration types (Figure 2). The expected increase in genome length from AAVs 2–6 (Table 1) was reflected by all types of calibration. It was possible to resolve rather small differences in genome length (smallest difference: 62 bases between the genomes of AAVs 5 and 6). Accuracy, defined as determined genome length/expected genome length × 100%, varies among the calibration types (Figure 2; Table 1). The nucleic acid calibrations returned the least accurate results with a general overestimation in genome length, especially when using dsDNA. In contrast, the protein calibration led to the most accurate determination of genome length in intact AAVs. Notably, for the protein and dsDNA calibration, the accuracy of genome length determination varies with genome length, and the slopes of the linear regressions are 0.8 and 1.3, respectively. For all calibration modes, the precision of genome sizing was high, with coefficient of variation (%CV) values below 2.6%.

Approach 2: Genome length determination on the basis of calibrations with AAVs of known genome length

Approach 2 focused on the genome length determination in intact AAVs on the basis of a calibration with AAV samples of various known genome lengths. The AAV calibrations either consider the contrast difference between empty and filled AAV peaks (referred to as Δ contrast; Figure 3A) or the absolute contrast values of the empty and filled AAV peaks (blue, referred to as Abs. contrast [filled/empty AAVs]; Figure 3B). Because of the large difference between the absolute contrast values of the empty and filled AAV peaks, the empty AAV's data point has as high degree of leverage, meaning a small error in the absolute contrast value might have a significant effect on the regression line. Consequently, in addition to the published approach from Wu et al.,[19] another calibration curve was generated based exclusively on the contrast values of filled AAVs (orange, referred to as Abs. contrast [filled AAVs]; Figure 3B). The slope of the calibration line changed substantially from -1.013×10^{-5} to -6.198×10^{-6} upon exclusion of the absolute contrast value of the empty AAV peak.

In the next step, the genome lengths of AAVs 2–6 were determined by applying these three calibration curves and plotted against the expected genome lengths (Figure 3C). In all cases, a positive correlation between determined and expected genome length was observed. In comparison with the Δ contrast calibration, when using both calibrations on the basis of the absolute contrast value, smaller differences in genome length could not be fully resolved. The Abs. contrast (filled/empty AAVs) calibration showed an overestimation of genome length for the AAV with a genome length of 3,793 bases, and the Abs. contrast (filled AAVs) calibration showed fluctuations in the upper genome length range. A resolution of the lowest difference in genome length of 62 bases (between AAVs 5 and 6) was provided only for the Δ contrast calibration. Although high accuracy for all types of calibration was observed, the Δ contrast calibration performed best, also showing high precision (Table 2). Similarly, repeating approach 2 with a high-molecular-weight standard instead

Table 1. Genome length of AAV samples 2–6 determined using four different calibration materials (approach 1), presented as mean of three measurement days with SD, %CV, and accuracy

	AAV 2	AAV 3	AAV 4	AAV 5	AAV 6
Expected genome length, bases	3,793	4,142	4,504	4,596	4,658
Protein calibration					
Genome length ± SD, bases	3,991 ± 91	4,271 ± 66	4,555 ± 94	4,641 ± 62	4,713 ± 125
Accuracy	105%	103%	101%	101%	101%
%CV	2.3%	1.5%	2.1%	1.3%	2.6%
RNA calibration					
Genome length ± SD, bases	4,278 ± 78	4,552 ± 81	4,910 ± 55	5,026 ± 56	5,114 ± 75
Accuracy	113%	110%	109%	109%	110%
%CV	1.8%	1.8%	1.1%	1.1%	1.5%
ssDNA plasmid calibration					
Genome length ± SD, bases	4,656 ± 85	4,954 ± 88	5,344 ± 60	5,470 ± 61	5,566 ± 82
Accuracy	123%	120%	119%	119%	119%
%CV	1.8%	1.8%	1.1%	1.1%	1.5%
dsDNA calibration					
Genome length ± SD, bases	5,606 ± 88	5,964 ± 107	6,390 ± 103	6,656 ± 109	6,769 ± 159
Accuracy	151%	147%	145%	146%	147%
%CV	1.8%	1.8%	1.1%	1.1%	1.5%

On each measurement day, 3 or 4 measurements were performed.

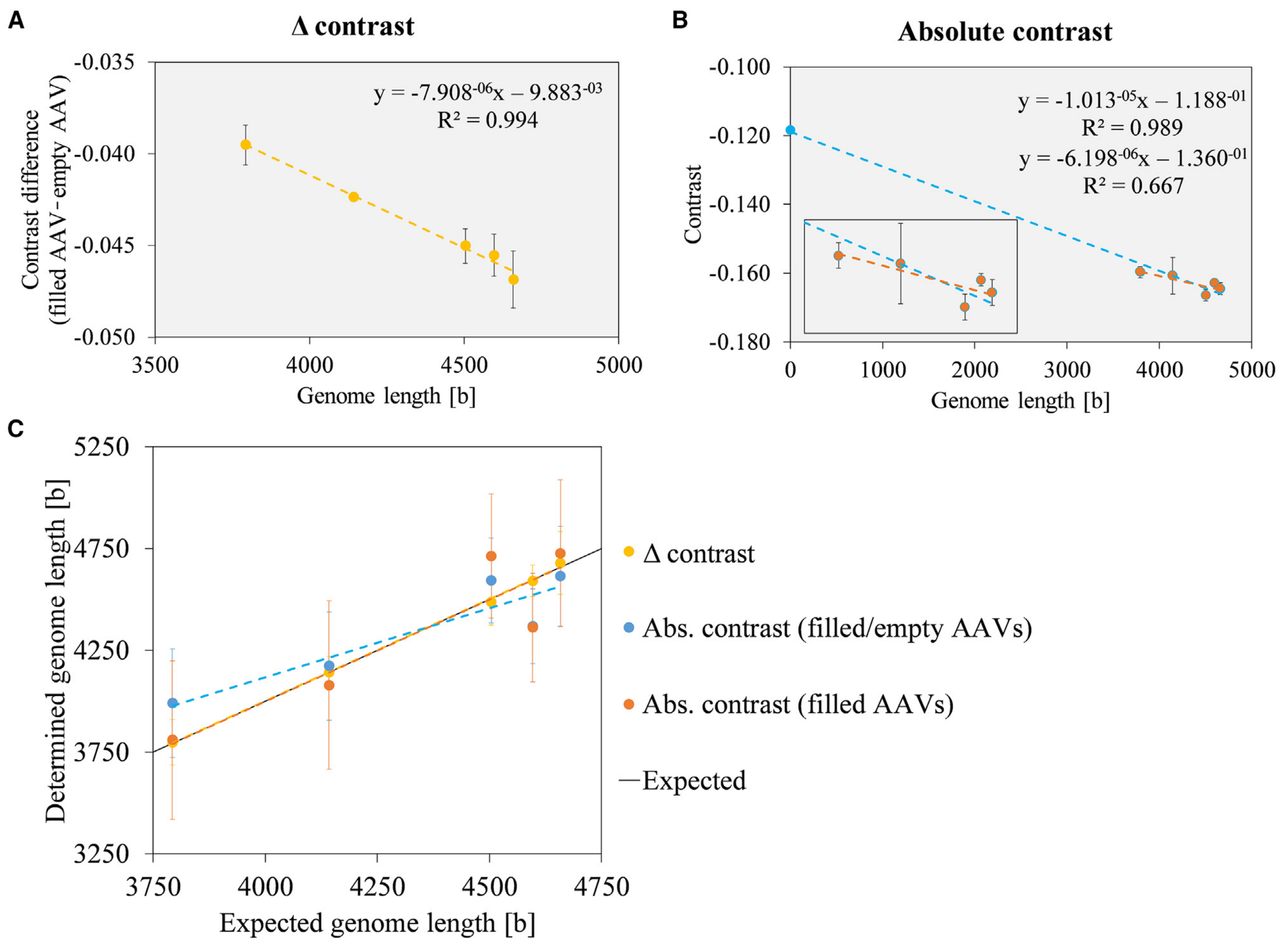

Figure 3. Approach 2

(A) Calibration curve on the basis of the contrast difference between empty and filled AAVs of known genome length (Δ contrast). (B) Calibration curve on the basis of the absolute contrast values of AAVs of known genome length including (Abs. contrast [filled/empty AAVs], blue) or excluding one empty AAV (Abs. contrast [filled AAVs], orange); inset graph shows the linear regression for the filled AAV values only. (C) Genome lengths (in bases) determined for five AAV samples using the three calibration types plotted against the expected genome lengths. Error bars represent SD (n = 3).

of the empty AAV peak as reference point revealed again a higher accuracy with higher precision in the genome length determination for the Δ contrast calibration in comparison with the Abs. contrast (filled AAVs) calibration (Figure S1; Table S2). Thus, it is assumed that the calculation of the contrast difference between the filled AAVs and a reference peak in the Δ contrast calibration compensates for the variability of the analysis workflow.

Approach 3: Genome length determination after genome release from AAV capsids

Heat-induced AAV disassembly. Heating of AAVs results in a destabilization of the AAV capsids and genome release, as demonstrated before by using atomic force microscopy and electron microscopy.[26,27] Correspondingly, AAV 7 of serotype 2 was heated at 65°C to induce genome release and analyzed with MP (Figure 4). This approach assumes that if the intact AAVs cannot be detected anymore after the disassembly procedure, the newly formed species with a defined peak at different, smaller contrast value corresponds to the released genome. After heat treatment, no intact AAVs could be detected (expected ratiometric contrast range about −0.1 to about −0.2; Figure 4C), but a species with a ratiometric contrast of about −0.041 appeared. Notably, next to the new peak, the histogram shows the presence of smaller fragments in the lower molecular mass region (ratiometric contrast value < −0.030). On the basis of a single-stranded nucleic acid calibration with either RNA or ssDNA plasmids the new species peak corresponded to a genome length of 4,407 ± 29 or 4,844 ± 31 bases, respectively, which is in the range of the expected genome length. However, this simple and fast heating workflow resulted in the generation of large particles, which interfered with the MP measurements. This was more pronounced when the genome release was performed at a higher temperature (75°C instead of 65°C) or a 3.5-fold higher AAV titer, as more particles formed hampering the detection of landing events (Figure 4A).

Table 2. Genome length of the AAV samples 2–6 determined using intact AAVs as calibration material (approach 2), presented as mean of the three measurement days with SD, %CV, and accuracy

Expected genome length, bases	AAV 2	AAV 3	AAV 4	AAV 5	AAV 6
	3,793	4,142	4,504	4,596	4,658
Δ contrast calibration					
Genome length ± SD, bases	3,798 ± 111	4,141 ± 80	4,488 ± 114	4,590 ± 77	4,679 ± 153
Accuracy	100%	100%	100%	100%	100%
%CV	2.9%	1.9%	2.5%	1.7%	3.3%
Abs. contrast (filled/empty AAVs) calibration					
Genome length ± SD, bases	3,990 ± 267	4,172 ± 266	4,594 ± 208	4,369 ± 183	4,614 ± 245
Accuracy	105%	101%	102%	95%	99%
%CV	6.7%	6.4%	4.5%	4.2%	5.3%
Abs. contrast (filled AAVs) calibration					
Genome length ± SD, bases	3,809 ± 391	4,079± 414	4,713 ± 305	4,363 ± 267	4,726 ± 361
Accuracy	100%	98%	105%	95%	101%
%CV	10.3%	10.2%	6.5%	6.1%	7.6%

On each measurement day, n=3 to 4 measurements were performed. (approach 3; n = 3 on a single measurement day), presented as mean with SD, %CV, and accuracy

AAV disassembly by heating in 1% SDS followed by SDS removal. Next, a second protocol for disassembly of the capsid was tested, which was based on heating in the presence of 1% sodium dodecyl sulfate (SDS).[28] Preliminary tests in MP showed that SDS concentrations above 0.001% in the sample lead to a textured image background, which impedes the proper detection of landing events and thus provides no data. In addition, SDS concentrations above 0.001% reduce the surface tension substantially, which makes sample application to the measurement wells challenging. Consequently, we removed the SDS from the heated AAV sample with microporous polystyrene beads.[29] The successful SDS removal was indicated by the restoration of surface tension and by the absence of a texturized background in the MP images, with the latter enabling the collection of data showing clear peaks for the species of interest.

Similarly to the solely heat-induced genome release, the workflow with SDS led to the generation of a new species with a ratiometric contrast of about −0.04, corresponding to a genome length of 4,376 ± 37 or 4,724 ± 40 bases on the basis of RNA or ssDNA plasmid calibration, respectively. Successful capsid disassembly was demonstrated by the absence of intact AAVs in the MP measurements (Figure 4C). Overall, SDS addition to the protocol showed two main advantages: (1) less formation of large aggregates, which interfere with MP measurements, and (2) substantially less fragments in the sample, with little landing events in the lower molecular mass region (ratiometric contrast value < −0.030) (Figure 4C).

This workflow was then applied to AAVs 2–6. In none of the samples, intact AAVs were detected and the intended new species corresponding to the released genome appeared (Figure 4D). When applying the single-stranded nucleic acid calibrations, the trend in the genome length agreed across all samples (Figure 4E). Small length differences down to 62 bases (AAV 5 vs. AAV 6) between the AAV genomes could be resolved. Moreover, accuracy values between 94% and 105% and %CV values below 1% for this dataset demonstrate the high accuracy and precision of this approach (Table 3).

DISCUSSION

In our study, we evaluated the potential of MP for AAV genome length determination and compared different sample preparation and calibration approaches for this purpose. The traditional methods for the verification of genome length in AAVs like denaturing agarose-gel electrophoresis, Southern blot, or SV-AUC are either very time or sample consuming, or they require very costly instrumentation. MP has recently emerged as an AAV analysis tool to assess the genome content in individual AAV capsids, thus providing information on the filled and empty fraction in an AAV sample. It has been shown that MP has the capability to resolve partially filled AAVs, but with slightly lower resolution in comparison with SV-AUC.[19] In this context, Wu et al.[19] observed a linear relationship between contrast values of filled AAVs and their genome length. On the basis of this finding, they proposed that MP may be applied to determine the length of the genome packaged into AAV vectors, but they did not perform any experiments in this direction.

To this end, we evaluated three MP-based approaches differing either in the reference material used for calibration (protein, ssDNA plasmids, dsDNA, RNA, or AAVs) or in AAV sample preparation (intact AAVs vs. disassembled AAVs). The success of the different approaches was evaluated on the basis of precision and accuracy of genome sizing, ease of handling, as well as material and time effort.

The most accurate results for genome length determination were achieved when analyzing intact AAVs with the help of an AAV calibration (approach 2). This approach is based on the assessment of peak contrast values of filled and empty AAVs. In comparison with the procedure proposed by Wu et al.,[19] which directly converts absolute contrast values to genome length for intact AAVs, the use of an empty AAV as internal reference to calculate contrast differences which are then converted into genome length improved repeatability and precision. Notably, all AAV-based calibrations determined the genome length very accurately at ±5%, which is within the error of 5% given by the instrument manufacturer.[30] In case of highly purified AVVs containing exclusively filled capsids and if an empty AAV sample is unavailable for spiking, a high-molecular-weight standard, once commercially available, might be used instead of the empty AAV for the Δ contrast calibration. In this situation, the contrast difference is calculated between the filled AAV and the high-molecular-weight standard, which again outperforms the calibration based on absolute ratiometric contrast values. Overall, all MP approaches, except for the two which use an AAV

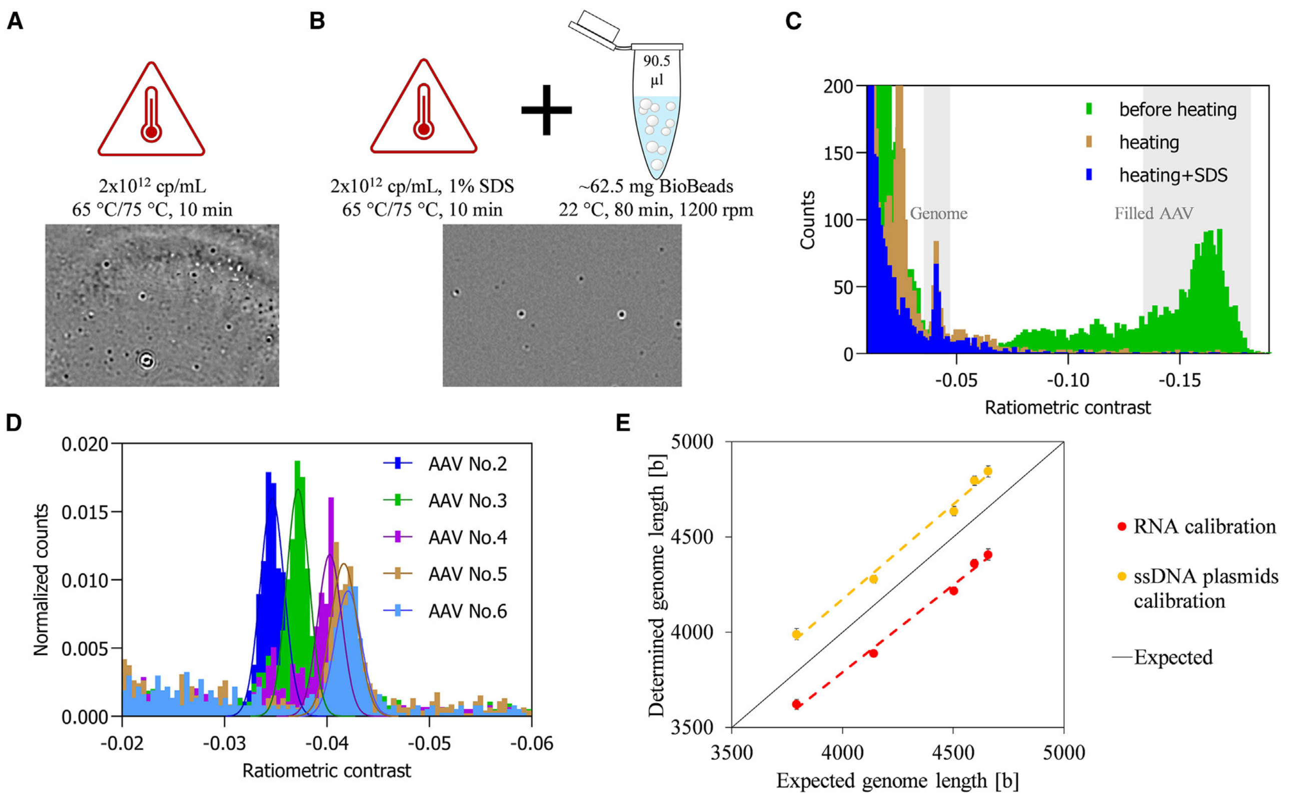

Figure 4. Approach 3

Workflows for genome release with exemplary ratiometric images of subsequent MP analysis for protocols on the basis of (A) heating or (B) heating in 1% SDS, followed by SDS removal. (C) Overlay of histograms from MP analyses of an AAV sample before and after processing with the two genome release workflows. (D) Overlay of histograms from MP analyses of five AAV samples with different genome lengths after genome release by heating in SDS, followed by SDS removal. (E) Determined genome lengths (in bases) plotted against the expected genome lengths for two different calibration materials. Error bars represent SD (n = 3).

calibration based on absolute ratiometric contrast values, were capable to resolve even the smallest genome-length difference of 62 bases (between AAVs 5 and 6 of our sample set). Nevertheless, the calibration based on absolute ratiometric contrast values of filled AAVs is of high value if the analyzed AAV sample only exhibits a small empty fraction and cannot be spiked with a reference molecule such as empty AAVs or high-molecular-weight standard. Notably, AAV-based calibration is constrained in practice because it requires the availability of a set of AAV preparations with distinct genome lengths. It should be mentioned that the presented approaches for genome length determination in intact AAVs compared individual samples containing AAVs of different genome size. Within a single AAV sample containing a mixture of AAV species of varying genome length, we estimate a minimum difference of ~400 bases to be required to allow a resolution of AAVs with differently sized genomes. For smaller differences in base number, those AAV species might not be resolved by MP and thus be detected in a single peak in the histogram of landing events. Consequently, a single Gaussian fit will be applied during data analysis, and MP will provide one single value of genome length for all AAVs contributing to the single peak.

The fastest approach for genome sizing is to convert the ratiometric contrast difference between the empty and filled population of an intact AAV sample into genome length using a protein calibration (approach 1). For this approach, the sample must contain an empty AAV population or empty AAV material needs to be available for spiking. Calibration with proteins gave the more accurate genome sizing results, with only a slight overestimation of genome length (101%–105%) and %CV values <2.6% in comparison with nucleic acid-based calibration types (ssDNA plasmids, dsDNA, RNA). Importantly, the calibration curves rely on the used class of molecules (e.g., protein, DNA), as the ratiometric contrast itself depends on the volume and the refractive index of the molecules.[19,23,24] In general, the genome length was overestimated when applying nucleic acid-based calibrations to convert the filled-empty AVV contrast differences to base number. This effect might be linked to different structural properties of nucleic acids in solution compared with the ssDNA genome confined in the AAV capsid.[27] The compaction of the AAV genome in the capsid might influence the ssDNA's light scattering properties. Additionally, we observed better accuracy with a protein-based calibration, which indicates that the MP scattering signal is more affected by the protein capsid than by the DNA content.

Table 3. Genome length of the AAV samples 2–6 determined after genome release by heating in SDS

Expected genome length, bases	AAV 2	AAV 3	AAV 4	AAV 5	AAV 6
	3,793	4,142	4,504	4,596	4,658
RNA calibration					
Genome length ± SD, bases	3,627 ± 27	3,888 ± 14	4,225 ± 15	4,362 ± 18	4,408 ± 23
Accuracy	96%	94%	94%	95%	95%
%CV	0.7%	0.4%	0.4%	0.4%	0.5%
ssDNA plasmid calibration					
Genome length ± SD, bases	3,996 ± 30	4,282 ± 18	4,644 ± 19	4,796 ± 19	4,846 ± 25
Accuracy	105%	103%	103%	104%	104%
%CV	0.7%	0.4%	0.4%	0.4%	0.5%

In contrast to approaches that analyze intact AAVs, length determination of the genome after its release (approach 3) requires no AAV material for calibration. Heat-induced release of the genome allowed rather accurate genome sizing using a single-stranded nucleic acid calibration, which is readily available. The simplicity of the workflow is appealing, but the formation of aggregates and fragments limits its quality and applicability. This problem could be resolved by a slightly more labor-intensive workflow, in which the sample is heated in the presence of 1% SDS and the surfactant subsequently removed. SDS favors the disassembly and solubilization of proteins, which reduces the presence of aggregates and fragments in the sample which interfere with the measurement. Depending on the calibration material, the genome release workflow with SDS resulted in a slight overestimation (ssDNA plasmid calibration) or underestimation (RNA calibration) of the genome length. This can again be attributed to chemical differences of the RNA ladder fragments or structural differences of the AAV's linear ssDNA genome compared with the plasmids' circular ssDNA. A linear ssDNA ladder would represent the ssDNA genome best, but such a ladder is, to the best of our knowledge, not commercially available. The determination of genome length after genome release benefits from a direct analysis of the isolated genome, meaning that the ratiometric contrast values are unaffected by the distribution of the AAV capsid mass. Thus, a narrow length distribution with high accuracy and precision (%CV values <1%) can be obtained. A drawback of genome sizing after release is the loss of information on the genomic payload on a single AAV level.

We have compiled a decision tree that provides guidance to select the most appropriate method for determining AAV vector genome length using MP depending on available sample material, calibration material, and time budget (Figure 5). Considering the promising results, further studies with AAV samples of different serotypes and genomic payloads (e.g., self-complementary genomes) should be performed to further reinforce the suitability of the established methods for genome length determination with MP.

MP is capable of rapidly assessing the genome length of AAV samples with minimal sample consumption, high accuracy, and good precision. Depending on the available AAV samples and reference materials, different MP-based approaches are suggested, each with benefits and limitations. The methods we developed are a reliable alternative to conventional methods, such as (capillary gel) electrophoresis, Southern blot, and AUC analysis. In particular, scientists already using MP for analysis of filled/empty capsid ratio of AAV preparations will be able to determine the genome length with minimal extra effort.

MATERIALS AND METHODS

Materials

Urease, thyroglobulin, poly-L-lysine (PLL) solution (0.01%), SDS solution (20% in water), isopropanol $\geq$99.5%, poloxamer 188, and hydrochloric acid 37% were purchased from Merck (Darmstadt, Germany). Nuclease-free water, PBS (10 mM phosphate, 138 mM NaCl, 3 mM KCl), low DNA mass ladder, and RiboRuler High Range RNA ladder were purchased from Thermo Fisher Scientific (Schwerte, Germany). M13 mp18 ssDNA and ΦX174 Virion DNA were obtained from New England Biolabs (Frankfurt am Main, Germany). High-molecular-weight standard of 3,643 kDa was provided by Refeyn (Oxford, UK). BioBeads SM-2 Adsorbent was purchased from Bio-Rad Laboratories (Hercules, CA). Ultra-purified water (Milli-Q IQ 7000 purification system; Merck, Darmstadt, Germany) was used to prepare all buffers and solutions. All chemicals and reagents were used as received. Molecules used for MP calibration were of a purity compatible with size exclusion chromatography (proteins), gel electrophoresis (nucleic acid ladders, ssDNA plasmids), or MP (high-molecular-weight standard).

AAV sample material

All AAV samples were purified by iodixanol gradient ultracentrifugation and formulated in PBS-MK (10 mM PBS, 1 mM $MgCl_2$, and 2.5 mM KCl_2 [pH 7.4]) with 0.001% (v/v) polysorbate-20 (PS20). Six AAV samples of the serotype AAV9, WT with expected genome lengths of 0 (empty capsid), 3,793, 4,142, 4,505, 4,596, and 4,658 bases (AAVs 1–6) and an AAV sample of serotype AAV2, WT with an expected genome length of 4,658 bases (AAV 7) were used. Capsid titers ranged from 5.43×10^{12} to 1.69×10^{15} capsid particles (cp)/mL and transgene titers from 2.56×10^{13} to 3.30×10^{14} viral genomes (vg)/mL. Table S1 in the supplemental information provides an overview of the AAV samples used in the study, including their serotype, transgene length, titers, and percentage of residual iodixanol. Titers and percentage of residual iodixanol were determined by a combination of UV/Vis spectroscopy, dynamic light scattering and static light scattering in a Stunner instrument (Unchained Labs, Pleasanton, CA). AAV samples were stored at −80°C after production and after thawing at 4°C up to four months.

Sample preparation

To obtain an appropriate count number at acceptable background noise in MP, all AAV samples were diluted in PBS-MK to a concentration between 1.00×10^{12} and 1.33×10^{12} cp/mL, except AAV sample 5, which was diluted to 5.00×10^9 or 7.12×10^9 cp/mL. Suitable

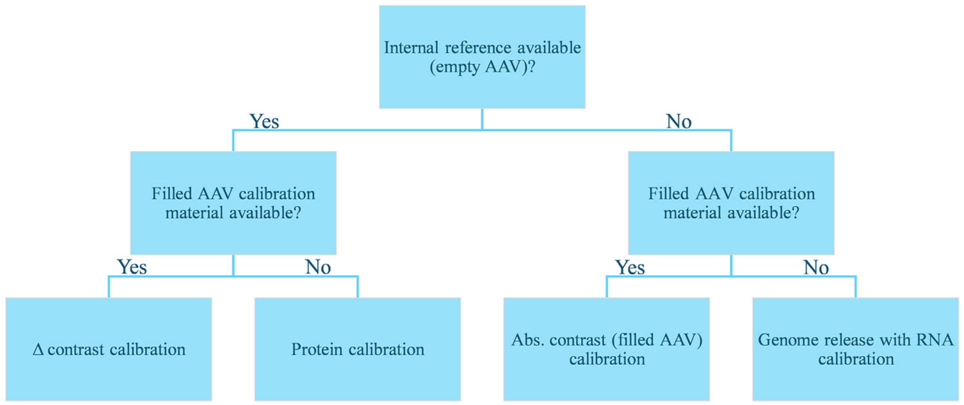

	Δ contrast calibration	Protein calibration	Abs. contrast (filled AAV) calibration	Genome release with RNA calibration
**Required calibration material**	4+ filled AAVs of known genome length, empty AAV	Urease, thyroglobulin, empty AAV	4+ filled AAVs of known genome length	RNA ladder
**# of samples for calibration**	4+	3	4+	1
**Calibration time**	3 h	2.5 h	3 h	45 min
**Workflow performance (accuracy, precision as %CV)**	100%, <3.3%	101% - 105%, <2.7%	95% - 105%, <10.3%	95% - 100%, <0.7%
**Drawbacks**	Filled and empty AAVs for calibration must be available; spiking of filled AAV samples with empty AAV before analysis	Slight overestimation of genome length; empty AAVs for calibration must be available; spiking of filled AAV samples with empty AAV before analysis	Filled AAVs for calibration must be available; lower genome length resolution	Loss of information on payload per single AAV capsid

Figure 5. Summary of the recommended genome length determination methods considering required calibration material, time, and effort for calibration, sizing accuracy, and precision

The decision tree should assist in finding the most suitable method for genome length determination on the basis of the available reference and calibration material. Internal reference refers to an empty AAV population, which can be used as reference to calculate the contrast difference between the empty and filled AAV population. The reference empty AAV population can be inherent to the AAV samples or can be spiked into the AAV samples. Once commercially available, empty AAVs might be replaced by a high-molecular-weight standard as reference.

capsid titers were reached when a one-minute MP analysis yielded peaks of approximately 300 counts for each AAV species of interest (filled or empty AAV or both). For approach 1, to ensure that both the empty and the filled AAV population are present in each sample, the empty AAV 1 sample was spiked to each AAV sample, resulting in a final titer of AAV 1 in the sample of 8.00×10^{11} cp/mL (AAV samples 2–4, 6, and 7) and 4.25×10^{9} cp/mL (AAV sample 5), respectively. When additionally testing the high-molecular-weight standard as reference point for the determination of contrast differences in approach 2, all AAV samples were spiked with the high-molecular-weight standard in a volume ratio between 1:1.2 and 1:3.8, with a higher portion of high-molecular-weight standard applied for samples containing a meaningful population of empty capsids. As described above, suitable spiking ratios of AAV 1 or high-molecular-weight standard was derived from the obtained count numbers. For approach 3, the genome length determination after genome release, two workflows were tested with the AAV 7 sample: (1) For heat-induced capsid destabilization, AAV 7 was first diluted in PBS to a concentration of 2×10^{12} cp/mL and subsequently heated at 65°C for 10 min while being shaken at 1,000 rpm on an Eppendorf thermomixer. The temperature was chosen on the basis of literature values for the melting temperature (T_m) of the AAV2 (capsid) serotype in PBS[31] and on previous publications on AAV genome release.[28] Additionally, a temperature of 75°C and a 3.5-fold higher AAV titer was tested. (2) For capsid disassembly by heating in 1% SDS followed by SDS removal, AAV 7 was first diluted in PBS with 1% SDS to a concentration of 2×10^{12} cp/mL and subsequently heated at 65°C for 10 min while being shaken at 1,000 rpm. Exploiting the adsorptive capacity of BioBeads SM2,[29] at least 62.5 mg of wetted beads were added per AAV sample (90.5 μL) to remove SDS. The mixture was incubated for 80 min at 1,200 rpm at room temperature (RT) and the supernatant collected. For AAV samples of serotype 9, the described workflows were maintained, but the heating temperature was increased to 75°C considering the higher T_m (77.0°C vs. 67.3°C) of the AAV9 WT compared with the AAV2 WT.[31]

Mass photometry

Data acquisition

Data were acquired at RT using a TwoMP mass photometer (Refeyn Ltd.) with a large field of view (AAV mode, 16.9 × 12.0 μm, 233 × 166 pixels), controlled using AcquireMP version 2.5.1 (Refeyn Ltd.). The required microscopy coverslips (24 × 50 mm; Thorlabs, Newton, NJ) were cleaned five times by sequential rinsing with isopropanol and water, followed by drying in a clean nitrogen stream. PLL-coated coverslips were prepared by sandwiching 7 μL of PLL solution between two cleaned coverslips. After incubation for 30 s at RT, the coverslips

were separated, and excess PLL was washed off by dipping the coverslips two times into a water-filled beaker and rinsing with water two times, before they were dried in a clean nitrogen stream. Measurement chambers were assembled by attaching a precut 2 × 3 well culture well silicon gasket (3 mm diameter × 1 mm depth; GBL103250-10 EA; Grace Bio-Labs, Bend, OR) to the prepared coverslips. After loading 14–18 μL buffer into a well to find the MP focus, a 2–6 μL sample was spiked into the same well to achieve a total volume of 20 μL and a final concentration of 2.5 nM thyroglobulin, 7.5 nM urease, 4.00 μg/mL RNA ladder, 1.96 μg/mL ΦX174 Virion DNA, 0.98 μg/mL M13 mp18 ssDNA, 1.96 μg/mL DNA ladder, or 6×10^8 to 2×10^{11} cp/mL for intact AAV samples. AAV samples after application of the genome release workflows were measured at a final concentration corresponding to 4.00×10^{11} to 6.00×10^{11} cp/mL in the intact samples. As previously mentioned, suitable amounts of applied sample were defined by a peak count value of ∼300 landing events. For each measurement, a video of 60 s was recorded. Each sample was analyzed at least three times ($n \geq 3$). Calibration proteins were measured on uncoated coverslips and calibration nucleic acids on PLL-coated coverslips and AAV samples with the corresponding coverslips.

Data analysis

Data were analyzed using DiscoverMP version 2.5.1 (Refeyn Ltd.). Histogram bandwidth was set to a molecular mass of 40 kDa and a genome length of 120 bases or 120 bp for analysis of intact AAVs or a genome length of 50 bases for the analysis of the released genome, respectively. Peaks in the histograms were fitted with a Gaussian distribution to extract peak contrast values and molecular mass or genome length, according to the applied calibration molecules. An overview of the molecular masses or base numbers of the calibration molecules is provided in Table S3.

For the protein-based contrast-to-mass calibration, peak contrast values were assigned to the known molecular masses of urease (272/545 kDa), thyroglobulin (670 kDa), and AAV 1 (empty AAV; 3,700 kDa). It should be mentioned that at the time of experiments on the basis of protein calibration, the high-molecular-weight standard (3,643 kDa) was not yet available as calibrant. When applying the protein calibration, the resulting mass difference between empty and filled AAVs in kilodaltons was converted into genome length in bases by applying a conversion factor of 309 kDa to 1,000 bases. For the dsDNA-based contrast-to-base-pair calibration, peak contrast values were assigned to the known DNA fragment lengths of 100, 200, 400, 800, 1,200, and 2,000 bp. The resulting genome-length difference between empty and filled AAVs in base pairs is converted to bases by multiplication by two (assuming that 1 base pair equals 2 bases). For the RNA-based contrast-to-bases calibration, the peak contrast values were assigned to the known RNA fragment lengths (200, 500, 1,000, 1,500, 2,000, 3,000, 4,000, and 6,000 bases). When ssDNA plasmids (ΦX174 Virion, 5,386 bases; M13 mp18, 7,249 bases) were applied for calibration, the calibration curve of contrast-to-bases consisted of the data points at the stated plasmid sizes and the zero point. From the contrast difference between the empty and filled AAV population or from the contrast of the released genome peak, genome lengths could then be determined in bases on the basis of the described contrast-to-bases conversions. AAV-based calibrations were established with the help of AAVs 2–6 (expected genome lengths of 3,793, 4,142, 4,504, 4,596, and 4,658 bases) and if required with AAV 1 (empty capsids) as reference point. For the first AAV calibration type, contrast differences between the empty and filled calibration AAV population were assigned to the known genome lengths. On the basis of the obtained calibration curve, the contrast differences between the empty and filled analyzed AAV population (Δ contrast) were then translated to bases. In the AAV calibration based on absolute ratiometric contrast values, peak contrast values of the empty and filled AAVs were assigned to their respective genome length and the genome length of the unknown filled AAV was derived directly from this contrast-to-bases calibration. The absolute contrast-based calibration curve was established once including and once excluding the empty AAV peak contrast value. Accuracy and precision (repeatability) of the genome length determination were assessed from the analysis of three independent experiments with at least triplicates ($n = 3$ or 4).

DATA AND CODE AVAILABILITY

The datasets generated during and/or analyzed in the present study are available from the corresponding author on reasonable request.

SUPPLEMENTAL INFORMATION

Supplemental information can be found online at https://doi.org/10.1016/j.omtm.2023.101162.

ACKNOWLEDGMENTS

This research did not receive any specific grant from funding agencies in the public, commercial, or non-for-profit sectors. The authors thank Kerstin Skokann (Ludwig-Maximilians-Universität [LMU]) for technical assistance with AAV vector production and Refeyn Ltd. for providing the high-molecular-weight standard.

AUTHOR CONTRIBUTIONS

C.H.: Conceptualization, Resources, Supervision, Project administration, Methodology, Writing - Original Draft; N.B.: Methodology, Investigation, Visualization, Writing - Review & Editing; C.H.: Conceptualization, Investigation, Resources, Supervision, Project administration, Writing - Review & Editing; A.H.: Conceptualization, Resources, Writing - Review & Editing; S.B.: Resources, Conceptualization, Writing - Review & Editing; S.M.: Resources, Conceptualization, Supervision, Writing - Review & Editing; W.F.: Conceptualization, Supervision, Writing - Review & Editing; T.M.: Conceptualization, Resources, Supervision, Writing - Review & Editing.

DECLARATION OF INTERESTS

The authors declare no competing interests.

REFERENCES

1. Wörner, T.P., Bennett, A., Habka, S., Snijder, J., Friese, O., Powers, T., Agbandje-McKenna, M., and Heck, A.J.R. (2021). Adeno-associated virus capsid assembly is divergent and stochastic. Nat. Commun. *12*, 1642.
2. Xie, Q., Bu, W., Bhatia, S., Hare, J., Somasundaram, T., Azzi, A., and Chapman, M.S. (2002). The atomic structure of adeno-associated virus (AAV-2), a vector for human gene therapy. Proc. Natl. Acad. Sci. USA *99*, 10405–10410.
3. Naso, M.F., Tomkowicz, B., Perry, W.L., and Strohl, W.R. (2017). Adeno-associated virus (AAV) as a vector for gene therapy. BioDrugs *31*, 317–334.
4. Samulski, R.J., and Muzyczka, N. (2014). AAV-mediated gene therapy for research and therapeutic purposes. Annu. Rev. Virol. *1*, 427–451.
5. Wang, D., Tai, P.W.L., and Gao, G. (2019). Adeno-associated virus vector as a platform for gene therapy delivery. Nat. Rev. Drug Discov. *18*, 358–378.
6. Muhuri, M., Maeda, Y., Ma, H., Ram, S., Fitzgerald, K.A., Tai, P.W., and Gao, G. (2021). Overcoming innate immune barriers that impede AAV gene therapy vectors. J. Clin. Invest. *131*, e143780.
7. Grimm, D., Kay, M.A., and Kleinschmidt, J.A. (2003). Helper virus-free, optically controllable, and two-plasmid-based production of adeno-associated virus vectors of serotypes 1 to 6. Mol. Ther. *7*, 839–850.
8. Clément, N., Knop, D.R., and Byrne, B.J. (2009). Large-scale adeno-associated viral vector production using a herpesvirus-based system enables manufacturing for clinical studies. Hum. Gene Ther. *20*, 796–806.
9. Matsushita, T., Elliger, S., Elliger, C., Podsakoff, G., Villarreal, L., Kurtzman, G.J., Iwaki, Y., and Colosi, P. (1998). Adeno-associated virus vectors can be efficiently produced without helper virus. Gene Ther. *5*, 938–945.
10. Lecomte, E., Tournaire, B., Cogné, B., Dupont, J.-B., Lindenbaum, P., Martin-Fontaine, M., Broucque, F., Robin, C., Hebben, M., Merten, O.-W., et al. (2015). Advanced characterization of DNA molecules in rAAV vector preparations by single-stranded virus next-generation sequencing. Mol. Ther. Nucleic Acids *4*, e260.
11. de la Maza, L., and Carter, B.J. (1980). Molecular structure of adeno-associated virus variant DNA. J. Biol. Chem. *255*, 3194–3203.
12. Wright, J.F. (2014). Product-related impurities in clinical-grade recombinant AAV vectors: Characterization and risk assessment. Biomedicines *2*, 80–97.
13. Schnödt, M., and Büning, H. (2017). Improving the quality of adeno-associated viral vector preparations: The challenge of product-related impurities. Hum. Gene Ther. Methods *28*, 101–108.
14. Dong, B., Nakai, H., and Xiao, W. (2010). Characterization of genome integrity for oversized recombinant AAV vector. Mol. Ther. *18*, 87–92.
15. Wu, Z., Yang, H., and Colosi, P. (2010). Effect of genome size on AAV vector packaging. Mol. Ther. *18*, 80–86.
16. Nass, S.A., Mattingly, M.A., Woodcock, D.A., Burnham, B.L., Ardinger, J.A., Osmond, S.E., Frederick, A.M., Scaria, A., Cheng, S.H., and O'Riordan, C.R. (2018). Universal method for the purification of recombinant AAV vectors of differing serotypes. Molecular therapy. Methods & clinical development *9*, 33–46.
17. Luo, J., Li, T., Wang, F., and Mollah, S. (2020). Technical Note: Genome Sizing Methodology for Genome Integrity Analysis of Adeno-Associated Viruses (AAV). https://www.sciex.com/content/dam/SCIEX/pdf/tech-notes/all/Brief-AAV-Genome-Integrity.pdf.
18. Barnes, L.F., Draper, B.E., Chen, Y.-T., Powers, T.W., and Jarrold, M.F. (2021). Quantitative analysis of genome packaging in recombinant AAV vectors by charge detection mass spectrometry. Mol. Ther. Methods Clin. Dev. *23*, 87–97.
19. Wu, D., Hwang, P., Li, T., and Piszczek, G. (2022). Rapid characterization of adeno-associated virus (AAV) gene therapy vectors by mass photometry. Gene Ther. *29*, 691–697.
20. Richter, K., Wurm, C., Strasser, K., Bauer, J., Bakou, M., VerHeul, R., Sternisha, S., Hawe, A., Salomon, M., Menzen, T., and Bhattacharya, A. (2023). Purity and DNA content of AAV capsids assessed by analytical ultracentrifugation and orthogonal biophysical techniques. Eur. J. Pharm. Biopharm. *189*, 68–83.
21. Hiemenz, C., Pacios-Michelena, A., Helbig, C., Vezočnik, V., Strebl, M., Nikels, F., Hawe, A., Garidel, P., and Menzen, T. (2023). Characterization of Virus Particles and Submicron-Sized Particulate Impurities in Recombinant Adeno-Associated Virus Drug Product. J. Pharm. Sci. *112*, 2190–2202.
22. Cole, D., Young, G., Weigel, A., Sebesta, A., and Kukura, P. (2017). Label-free single-molecule imaging with numerical-aperture-shaped interferometric scattering microscopy. ACS Photonics *4*, 211–216.
23. Young, G., Hundt, N., Cole, D., Fineberg, A., Andrecka, J., Tyler, A., Olerinyova, A., Ansari, A., Marklund, E.G., Collier, M.P., et al. (2018). Quantitative mass imaging of single biological macromolecules. Science *360*, 423–427.
24. Li, Y., Struwe, W.B., and Kukura, P. (2020). Single molecule mass photometry of nucleic acids. Nucleic Acids Res. *48*, e97.
25. Lai, S.-H., Tamara, S., and Heck, A.J.R. (2021). Single-particle mass analysis of intact ribosomes by mass photometry and Orbitrap-based charge detection mass spectrometry. iScience *24*, 103211.
26. Bernaud, J., Rossi, A., Fis, A., Gardette, L., Aillot, L., Büning, H., Castelnovo, M., Salvetti, A., and Faivre-Moskalenko, C. (2018). Characterization of AAV vector particle stability at the single-capsid level. J. Biol. Phys. *44*, 181–194.
27. Horowitz, E.D., Rahman, K.S., Bower, B.D., Dismuke, D.J., Falvo, M.R., Griffith, J.D., Harvey, S.C., and Asokan, A. (2013). Biophysical and ultrastructural characterization of adeno-associated virus capsid uncoating and genome release. J. Virol. *87*, 2994–3002.
28. Oyama, H., Ishii, K., Maruno, T., Torisu, T., and Uchiyama, S. (2021). Characterization of adeno-associated virus capsid proteins with two types of VP3-related components by capillary gel electrophoresis and mass spectrometry. Hum. Gene Ther. *32*, 1403–1416.
29. Lacapère, J.J., Delavoie, F., Li, H., Péranzi, G., Maccario, J., Papadopoulos, V., and Vidic, B. (2001). Structural and Functional Study of Reconstituted Peripheral Benzodiazepine Receptor. Biochem. Biophys. Res. Commun. *284*, 536–541.
30. Refeyn. Data Sheet: Refeyn Two Mass Photometer. https://www.refeyn.com/twomp-mass-photometer.
31. Bennett, A., Patel, S., Mietzsch, M., Jose, A., Lins-Austin, B., Yu, J.C., Bothner, B., McKenna, R., and Agbandje-McKenna, M. (2017). Thermal stability as a determinant of AAV serotype identity. Mol. Ther. Methods Clin. Dev. *6*, 171–182.

OMTM, Volume *31*

Supplemental information

Genome length determination

in adeno-associated virus vectors

with mass photometry

Cornelia Hiemenz, Nadine Baumeister, Constanze Helbig, Andrea Hawe, Sabrina Babutzka, Stylianos Michalakis, Wolfgang Friess, and Tim Menzen

Table S1. List of AAV samples (cp, capsid particle; vg, viral genome; WT, wild-type). The expected genome length corresponds to the used transgene size. Transgene size was confirmed by sanger sequencing (Eurofins Genomics). Transgene titer was determined by qRT-PCR with ITR-specific primers on a StepOnePlus Real-Time PCR system (Applied Biosystems, Thermo Fisher Scientific). Standard curves were established using ITR-specific primers as described before [1], [2]. Capsid titer as well as content of residual iodixanol were determined by using the Stunner instrument (Unchained Labs); (*) Note that the high residual iodixanol content in the AAV No. 5 sample most likely led to an underestimation of capsid titer.

**No.**	**Serotype**	**Transgene size [b]**	**Transgene titer [vg/mL]**	**Capsid titer [cp/mL]**	**Residual iodixanol [% (v/v)]**
**1**	AAV9 WT	0 (empty capsid)	-	2.91×10^{14}	12.0
**2**	AAV9 WT	3793	2.55×10^{14}	8.70×10^{14}	3.5
**3**	AAV9 WT	4142	3.14×10^{14}	9.62×10^{14}	4.2
**4**	AAV9 WT	4504	3.30×10^{14}	1.69×10^{15}	5.4
**5**	AAV9 WT	4596	6.88×10^{13}	5.43×10^{12}*	28.0
**6**	AAV9 WT	4658	6.73×10^{13}	3.50×10^{14}	13.0
**7**	AAV2 WT	4658	2.56×10^{13}	1.81×10^{14}	10.0

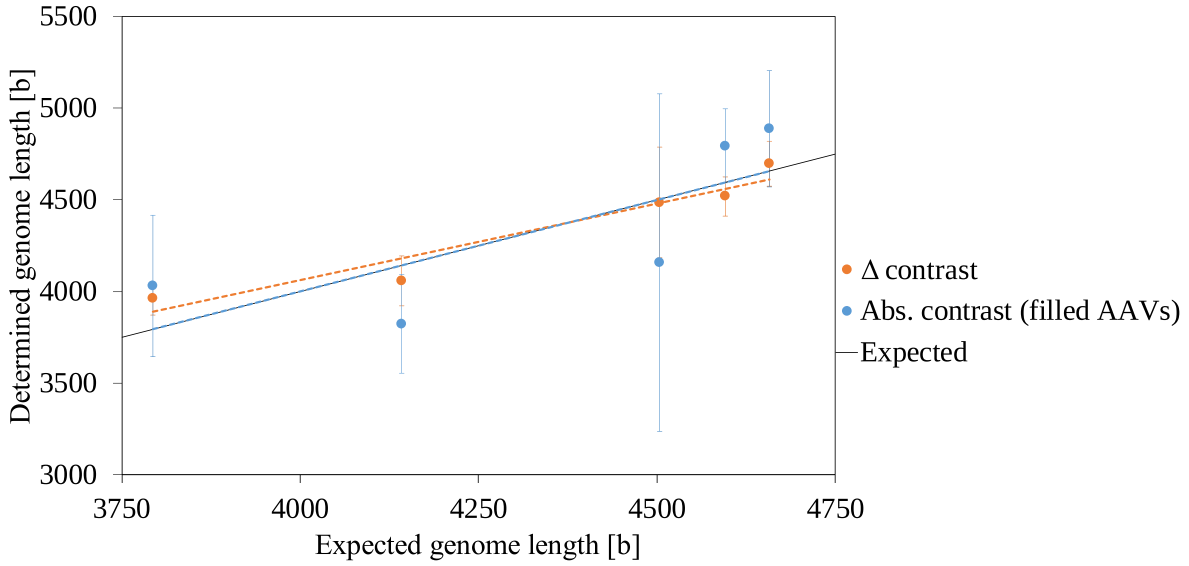

Figure S1. Genome length determination according to approach 2, when High Molecular Weight standard was used as reference point: Genome lengths (in bases) determined for five AAV samples using either a calibration curve based on the contrast difference between High Molecular Weight standard and filled AAVs of known genome length (Δ contrast) or a calibration curve based on the absolute contrast values of AAVs of known genome length (Abs. contrast (filled AAVs)); Error bars represent standard deviation (n=3).

Table S2. Genome lengths of the AAV samples No. 2-6 determined using intact AAVs as calibration material (approach 2), presented as mean of the three measurement days with SD, %CV and accuracy. On each measurement day, n=3 to 4 measurements were performed. In this case, the ratiometric contrast difference (Δ contrast) was calculated between the filled AAV and the High Molecular Weight standard (instead of empty AAV).

		**AAV No. 2**	**AAV No. 3**	**AAV No. 4**	**AAV No. 5**	**AAV No. 6**
**Expected genome length [b]**		3793	4142	4504	4596	4658
**Δ contrast calibration**	Genome length ± SD [b]	3963 ± 91	4057 ± 137	4486 ± 300	4518 ± 107	4697 ± 123
	Accuracy	104%	98%	100%	98%	101%
	%CV	2.3%	3.4%	6.7%	2.4%	2.6%
**Abs. contrast (filled AAVs) calibration**	Genome length ± SD [b]	4030 ± 387	3823 ± 271	4156 ± 921	4794 ± 203	4887 ± 317
	Accuracy	106%	92%	92%	104%	105%
	%CV	9.6%	7.1%	22.2%	4.2%	6.5%

Table S3. Molecular masses or genome lengths of the molecules used for MP calibration; MW marker, molecular weight marker. *When ssDNA plasmids were applied for calibration, the calibration curve was fitted through the zero point.

**Protein-based calibration curve**				
Molecule	Urease (monomer)	Urease (dimer)	Thyroglobulin	AAV No. 1 (empty capsid)
Molecular mass [kDa]	272	545	670	3700

**RNA-based calibration curve**								
NW marker	RiboRuler High Range RNA ladder							
Fragment length [b]	200	500	1000	1500	2000	3000	4000	6000

**ssDNA-based calibration curve**			
Plasmid	Zero point*	ΦX174 Virion	M13 mp18
Genome length [b]	0	5386	7249

**dsDNA-based calibration curve**						
MW marker	Low DNA mass ladder					
Fragment length [b]	100	200	400	800	1200	2000

**AAV-based calibration curves**					
AAV	AAV No. 2	AAV No. 3	AAV No. 4	AAV No. 5	AAV No. 6
Genome length [b]	3793	4142	4504	4596	4658

REFERENCES

[1] C. Aurnhammer *et al.*, "Universal real-time PCR for the detection and quantification of adeno-associated virus serotype 2-derived inverted terminal repeat sequences," *Hum. Gene Ther. Methods*, 2012, doi: 10.1089/hgtb.2011.034.

[2] S. D'Costa *et al.*, "Practical utilization of recombinant AAV vector reference standards: focus on vector genomes titration by free ITR qPCR," *Mol. Ther. - Methods Clin. Dev.*, vol. 3, no. January, p. 16019, 2016, doi: 10.1038/mtm.2016.19.
